# Triple-negative breast cancer: multipronged approach, single-arm pilot phase II study

**DOI:** 10.1002/cam4.3

**Published:** 2012-05-31

**Authors:** Francesco Recchia, Giampiero Candeloro, Giovambattista Desideri, Stefano Necozione, Cornelia O C Recchia, Vincenzo Cirulli, Silvio Rea

**Affiliations:** 1 Oncology Unit, Civilian HospitalAvezzano, Italy; 2 Carlo Ferri FoundationMonterotondo, Rome, Italy; 3 Department of Internal Medicine and Public Health, University of L'AquilaItaly; 4 Psychological Oncology, Civilian HospitalAvezzano, Italy; 5 Department of Medicine, University of WashingtonSeattle, Washington; 6 Department of Experimental Medicine, University of L'AquilaItaly

**Keywords:** Concurrent chemotherapy and radiation therapy, high-dose chemotherapy, platinum analogues, triple-negative breast cancer

## Abstract

Anthracyclines (A) and taxanes (T) are standard first-line chemotherapy agents for patients with advanced breast cancer. Platinum analogues have also shown activity in the triple-negative breast cancer (TNBC) histology, but clinical data are limited. Here we report the long-term follow-up of a phase II study on TNBC treated with a combined modality therapy, including induction with AT, cyclophosphamide, methotrexate, and 5-fluorouracil (CMF) with concurrent radiation therapy, and a dose-dense consolidation chemotherapy (HDCT) with carboplatin (CBDCA), ifosfamide (IFX), etoposide (VP-16). Patients' median age was 44 years, with 73% premenopausal. Epirubicin 75 mg/m^2^ and docetaxel 75 mg/m^2^ were administered to 70 patients with TNBC: as neoadjuvant and adjuvant therapy to 12 and 58 patients, respectively. Postoperative radiation therapy, 5000 cGy, was delivered, synchronous with triweekly CMF. After radiation therapy, two courses of HDCT with CBDCA, IFX, VP-16, were given, with hematological growth factors. After a median follow-up of 81 months, all patients were evaluable for toxicity and response. Most important toxicity were grade 3 skin reaction and grade 4 hematological in 3% and 31% of patients, respectively. Pathological complete response was observed in 25% of patients receiving preoperative chemotherapy. Treatment failures were as follows: eight visceral, four contralateral breast cancer, four locoregional, and one leukemia. Five-year progression-free survival and overall survival rate were 78% and 91%, respectively. Induction chemotherapy, followed by chemoradiation therapy and HDCT, provides a prolonged disease-free period and a significant increase in overall survival in TNBC, with an acceptable toxicity profile.

## Introduction

Basic knowledge on mechanisms causing breast cancer progression has driven significant progresses in its treatment, with the introduction of more sophisticated hormonal and targeted therapies. Unfortunately triple-negative breast cancer (TNBC) is a biological entity that lacks estrogen receptors (ER), progesterone receptors (PGR), and human epidermal growth factor receptor 2 (HER2) [[1, 2]]. These phenotypic traits render TNBC unresponsive to some of the most effective biologic therapies now available. Based on gene expression profiling and immunohistochemical morphometric assessments, TNBCs, which account approximately for ∼13% of all breast cancers, have been suggested to be synonymous with basal-like tumors [1, 3–6]. Patients with TNBC have a grim prognosis with a short progression-free survival (PFS) and overall survival (OS) [2, 7]. Although several studies are defying the role of biological agents such as poly adenosine diphosphate-ribose polymerase (PARP) inhibitors in the management of TNBC [8], chemotherapy, whose benefits have been clearly demonstrated in multiple studies, remains the mainstay for the treatment of these patients in the neoadjuvant, adjuvant, and metastatic disease setting [9–11]. Anthracyclines and taxanes are considered the most active agents in the treatment of breast cancer [12, 13]. Docetaxel has been shown, in phase II studies, to induce responses in over 50% of patients with anthracycline-resistant breast cancer [14]. Moreover, the National Surgical Adjuvant Breast and Bowel Project Protocol B-27 has demonstrated that the addition of four cycles of preoperative docetaxel after four cycles of preoperative adriamycin cyclophosphamide significantly increased clinical and pathological response rates for operable breast cancer [15]. The importance of radiation therapy (XRT) in the treatment of breast cancer has been demonstrated in a randomized study: After 15 years of follow-up, the women assigned to chemotherapy plus XRT had a 33% reduction in the rate of recurrence and a 29% reduction in mortality from breast cancer, as compared with the women treated with chemotherapy alone [16]. Furthermore, another study, conducted in high-risk premenopausal women with breast cancer, showed that the addition of postoperative irradiation to mastectomy and adjuvant chemotherapy reduced locoregional recurrences and prolonged survival [17]. An additional advantage of administering radiation therapy synchronously with chemotherapy is the shortening of the overall duration of treatment without a substantial increase in toxicity [18].

At the time our study was undertaken, there was a limited experience in the treatment of breast cancer with platinum analogues [19, 20]. Nevertheless, since January 1991 we had performed a study of carboplatin (CBDCA), cyclophosphamide (CTX), etoposide (VP-16), in the treatment of patients with metastatic breast cancer progressing after anthracyclines [21]. Sixty-six percent of patients obtained a clinical benefit from this regimen with a substantial palliation of symptoms. Encouraged by these results, we designed a new study with a dose-dense regimen using the same drugs, supported by hematological growth factors, as salvage chemotherapy in a cohort of patients with advanced and refractory solid tumors [22]. A significant activity of this chemotherapy could be observed in the 23 patients with anthracycline-resistant advanced breast cancer. In addition, due to the phenotypic and molecular similarities existing between TNBC and BRCA-associated breast cancer we reasoned that both cancers may share sensitivity to platinum analogues. Based on these earlier experiences, in June 2001 we designed a new study and opened the recruitment of patients with ER, PGR, and fluorescence in situ hybridization (FISH) negative breast tumors, and herein we report the long-term follow-up.

## Patients and Methods

### Eligibility criteria

The study recruited biopsy-proven, previously untreated patients with large (T2–T4) ER negative (ER−), PGR negative (PGR−), HER2-negative breast cancer, aged 18–70 years, with an Eastern Cooperative Oncology Group (ECOG) performance status <2, and a life expectancy of at least 12 weeks. The patients, not pregnant or lactating, had to have an adequate hematological reserve and hepatic and renal function, documented by a WBC count >3000/mm^3^, absolute neutrophil count >1500/mm^3^, hemoglobin level >9.0 g/dL, platelets >100,000/mm^3^, serum bilirubin <1.5 mg/dL, aspartate aminotransferase, and alanine aminotransferase <4 times the upper limit of normal and normal cardiac and renal functions (ejection fraction >50%, serum creatinine <1.4 mg/dL). Patients with additional malignancies, other than curatively treated skin and cervical cancer or with active cardiovascular disease, were excluded. The protocol was approved by the Ethical Committee of the Civilian Hospital of Avezzano, Italy, and of the other participating institutions, and written informed consent was obtained from each patient.

### Pretreatment evaluation

A complete staging workup was carried out, including medical history, physical examination, electrocardiogram, and bidimensional echocardiography, mammography, chest X-ray, liver ultrasonography, and radionuclide bone scan. A computed tomography or magnetic resonance imaging of the brain was also carried out in case of suggestion of brain involvement. Baseline laboratory studies included complete blood counts (CBCs), liver and renal function tests, estradiol, progesterone, follicle-stimulating hormone (FSH), luteinizing hormone (LH), carcino-embryonic antigen (CEA), and carbohydrate antigen (CA 15-3). A core biopsy of the breast tumor was also performed along with immunohistochemical assessment of hormone receptors, Ki-67, HER2. In case of HER2 (+), FISH was performed to determine HER2 positivity. Tumors were classified as ER+ or PGR+ if staining was present in >1% or more of tumor nuclei. The Ki-67 cut point of 13% was used to designate a tumor as high proliferation.

### Treatment

An intensive psychological support that improved adherence to treatment was implemented by one of the authors (C. O. C. R.). Fifty-one (73%) of the 70 patients were premenopausal: they received a leuteinizing hormoneion [23]. The six patients with inflammatory breast cancer and the six patients with T4 tumors had a core biopsy to obtain tumor tissue for study. A radio-opaque clip was placed in the tumor bed of T4 tumors. These 12 patients received the induction chemotherapy preoperatively, while the 58 patients with T3 and T2 tumors had the same chemotherapy postoperatively, every 3 weeks for four courses. The chemotherapy regimen consisted of epirubicin 75 mg/m^2^ and docetaxel 75 mg/m^2^, both given over 2 hours on day 1. Premedication with dexamethasone at a dose of 8 mg was given 12, 6, and 1 hour before docetaxel administration and then twice a day for 4 days after chemotherapy. Breast-sparing surgery when feasible, or modified radical mastectomy together with standard level I and II axillary lymph node dissection, was carried out 3 weeks after the end of chemotherapy for patients with T4 or inflammatory tumors and as initial treatment to the 58 patients with T2–T3 tumors. XRT was delivered to the 26 patients with radical mastectomy and to the 44 patients with quadrantectomy at the dose of 5000 cGy, 200 cGy/fraction, 5 fractions/week, to the chest wall after mastectomy or to the residual breast after breast-conserving surgery, and at the apex of the axilla and supraclavicular lymph nodes. A boost of 1000 cGy was delivered to the tumor bed. XRT was given synchronous with six courses of triweekly cyclophosphamide 600 mg/m^2^, methotrexate 40 mg/m^2^, 5-fluorouracil 600 mg/m^2^ (CMF). One month after the end of chemoradiation therapy, two courses of dose-dense chemotherapy (HDCT) with CBDCA, area under the concentration curve (AUC) = 7, VP-16 400 mg/m^2^, ifosfamide, and uromitexan 6000 mg/m^2^, were delivered, over 3 days, supported by glycosilated recombinant granulocyte colony-stimulating factor (G-CSF) 300 μg/day. Chemotherapy was delivered at full doses if absolute neutrophil count (ANC) >1500 mm^3^ and platelets >100,000 mm^3^. For ANC between 1000 and 1499 mm^3^, and/or platelets between 75,000 and 99,000 mm^3^, chemotherapy was administered at doses reduced to 50%. In presence of lower ANC or platelet values, treatment was omitted.

### Toxicity and response evaluation criteria

Toxicity was evaluated according to the National Cancer Institute—Common Toxicity Criteria version 3. A CBC and chemistry were checked weekly in all patients and daily in case of grade 4 leukopenia. A bidimensional echocardiogram was carried out at completion of treatment and every 6 months thereafter for 3 years. After chemotherapy, tumor response was assessed according to standard World Health Organization criteria [24]. Medical assessment, mammography, and breast sonograms were carried out to evaluate tumor response in both breast and axilla. Pathological analysis of response took into account, the amount of residual epithelial neoplastic cells in the tumor mass, the mitotic index, and the location of malignant component (invasive vs. intraductal) [25]. Pathological complete response (pCR) was defined as no presence of tumor or microscopic disease in the breast samples, and resected axillary lymph nodes.

### Statistical analysis

The number of patients required for the study was calculated according to a Simon minimax design [26]. The first stage required at least 15 of 23 patients to exhibit a confirmed 5-year survival rate of 77% (P1), to rule out an undesirably low response probability of 0.62 (P0), with a 5% probability of accepting a poor agent (α= 0.05) and a 20% probability of rejecting a good agent (β = 0.20). In the second stage, a total of 70 assessable patients were to be added if 49 or more patients showed a 77% of the 5-year survival rate. The PFS was defined as the time between the start of adjuvant therapy to any relapse and the appearance of a second primary cancer or death, whichever occurred first. The OS was measured from study entry to death, or July 2011 for censored patients. Statistical analysis of PFS and OS was performed using the Kaplan–Meier method [27]. All comparisons were performed using Pearson's χ^2^ contingency table analysis. Statistical analysis was performed with SAS statistical software (version 8.12, 2000; SAS Institute Inc, Cary, NC).

### Patients

From July 2001 to April 2007, 70 consecutive cases with triple-negative large breast cancer were entered in the study at the Civilian Hospital of Avezzano, at the University of L'Aquila and at the Foundation “Carlo Ferri”. Table 1 summarizes the main patient characteristics. Twenty-five patients showed T2 and 33 T3 disease, six patients had T4 and six had inflammatory tumors, respectively. A total of 206 positive axillary nodes were observed. The distribution of nodes was as follows: nine patients had a mean number of 13.1 positive axillary nodes (range 10–20) (pN3), two patients had a mean number of 4.5 positive axillary nodes (range 4–5) (pN2), 41 patients had a mean of 1.5 positive axillary node (range 1–3) (pN1), while 18 patients (26%) had negative axillary nodes (pN0). The mean T size at diagnosis was 4 cm. Tumor grade was intermediate (G2) or high (G3) in 26 and 44 patients, respectively. Mean Ki-67 was 55% ± 2.7% (range 20%–95%).

**Table 1:** Patient and tumor characteristics

Characteristics	No.	%
No. of patients	70	100
Age (years)		
Median	44	
Range	26–70	
Menopausal status		
Pre	51	73
Post	19	27
Tumor histology		
Ductal infiltrating	57	81
Lobular infiltrating	9	13
Undifferentiated	4	6
T status		
T2	25	36
T3	33	48
T4	6	8
Inflammatory	6	8
N status		
N0	18	26
N1	41	58
N2	2	3
N3	9	13
Grading		
G1–G2	26	37
G3	44	63
KI-67		
<20%	4	6
>20%	66	94
Clinical stage		
II	47	67
III	23	33
Inflammatory	6	9
Primary chemotherapy	12	18
Surgery		
Mastectomy	26	37
Quadrantectomy	44	63

## Results

### Response and survival

**Figure 1:**
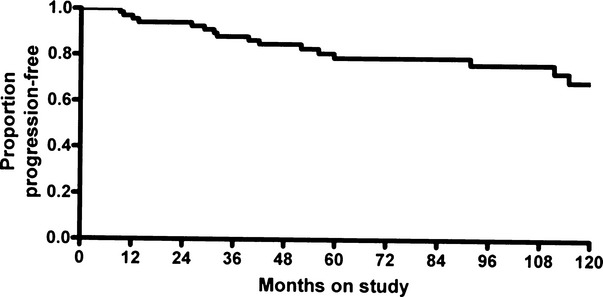
Progression-free survival (PFS) of all patients. Events 17 (24%), censored 53 (76%). Three-year PFS rate (88%, 95% CI 80–93). Five-year PFS rate (78%, 95% CI 68–85).

**Figure 2:**
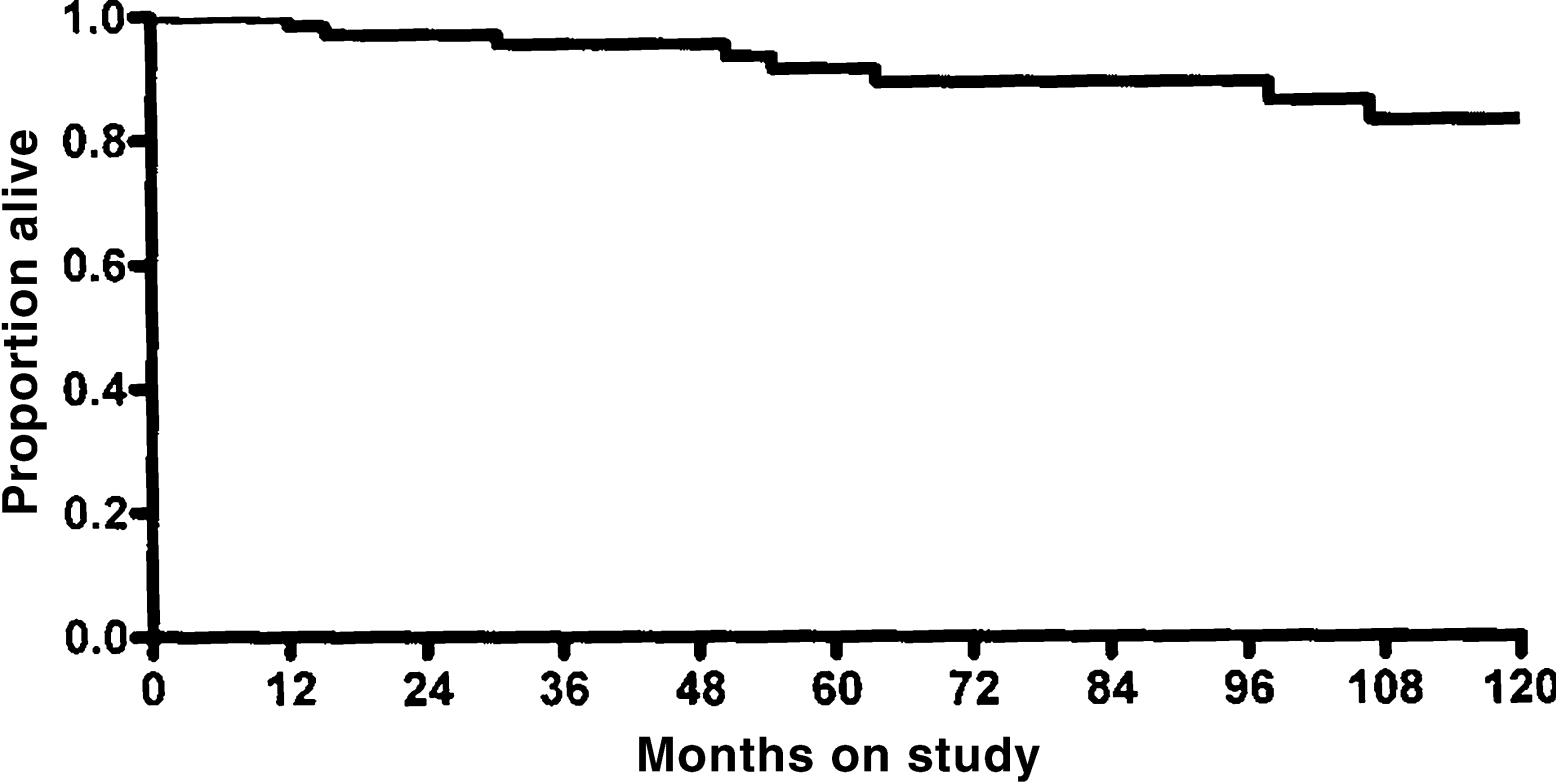
Overall survival (OS) of all patients. Events 8 (11%), censored 62 (89%). Three-year OS rate (95%, 95% CI 88–98). Five-year OS rate (91%, 95% CI 83–95).

All the patients completed the planned protocol. Analysis was performed on the basis of intention to treat. After a median follow-up of 81 months (minimum 24.2 months), 53 patients (76%) were progression free and 62 patients (89%) were alive. Median PFS was not reached yet, and 5-year PFS and OS rate were 78% and 91%, respectively, while 10-year PFS and OS rate were 64% and 83%, respectively (Figs 1 and 2). Objective response observed in the patients treated with neoadjuvant chemotherapy was as follows: 25% pCR, 67% partial responses, 8% stable disease. No patient had disease progression. Surgery was performed 3 weeks after the fourth course of neoadjuvant chemotherapy. Partial mastectomy was performed in 44 (63%) patients, while modified radical mastectomy was accomplished in 26 (37%) patients. Two patients with inflammatory breast cancer and two patients with T4 tumors showed a pCR in the tumor and in the axilla. Full axillary dissection was accomplished in all patients. Four patients developed recurrence in the controlateral breast: they underwent modified radical mastectomy. Three of these patients are presently progression free, while one of them developed systemic recurrence and died of the disease. Eight patients had visceral recurrence: four in the lung, three in the liver, and one in the soft tissues. One patient developed acute leukemia and died of the disease 5 months later. One patient with pathological complete response refused the last chemotherapy cycle with carboplatin, cyclophosphamide, and etoposide.

### Toxicity

Table 2 summarizes the toxicity data observed in the 70 patients entered into the study. No treatment-related deaths or life-threatening events occurred throughout the duration of the study.

**Table 2:** Toxicity

	Grade (NCI CTC version 3)									
	
	1		2		3		4		Total	
					
	No.	%	No.	%	No.	%	No.	%	No.	%
Hematologic										
Leucopenia	12	17	27	39	12	17	6	9	57	81
Neutropenia	10	14	15	21	16	23	21	30	62	89
Thrombocytopenia	3	4	2	3	1	1	6	9	12	17
Anemia	11	16	13	19	0	0	0	0	24	34
Infection	0	0	0	0	3	4	0	0	3	4
Gastrointestinal										
Oral	15	21	20	29	5	7	0	0	40	57
Nausea and vomiting	34	49	13	19	2	3	0	0	49	70
Diarrhea	21	30	12	17	0	0	0	0	33	47
Hepatic	10	14	5	7	2	3	0	0	17	24
Cardiac	5	7	5	7	0	0	0	0	10	14
Alopecia	10	14	60	86	0	0	0	0	70	100

Thirty-seven (53%) patients showed grade 3–4 neutropenia. Neutrophils fell below 0.5 × 10^9^/L in 21 patients, with only three episodes of febrile neutropenia. Seven patients showed grade 3–4 thrombocytopenia, but no platelet transfusion was required.

Alopecia was almost universal. Grade 3 skin reaction was observed in two patients during radiation therapy synchronous with chemotherapy. Among the other non-hematological side-effects, emesis, fatigue, loss of appetite, and mucositis were the most common. In particular, 70% of patients complained of nausea/vomiting, but only in few cases was it severe. Half of patient patients suffered from grade 1 diarrhea. Severe stomatitis was observed in five (7%) patients, while no episodes of grade 3 peripheral neuropathy were observed. Musculoskeletal symptoms like transient arthralgias and myalgias occurred in a total of 32 (45%) patients, but they were severe in only two cases, and generally responded well to acetominofen. In the majority of cases, these symptoms were related to G-CSF administration.

Cardiac toxicity was almost absent. Only five patients developed a decline in left ventricular ejection fraction (LVEF) by >10%, but this remained asymptomatic and no treatment was required. Finally, seven episodes of transient increase of fivefold of the aspartate aminotransferase (AST) and alanine aminotransferase (ALT) serum levels were observed, without any clinical sign of liver dysfunction.

## Discussion

Recent progress in medical science has led to a sophisticated classification of breast carcinomas, based on variations in gene expression patterns derived from cDNA microarrays. This classification, distinguishing breast cancers into basal type (triple negative), luminal, and HER2/neu, has been correlated with clinical outcome and response to therapy [1–5]. However, currently available immunohistochemical markers can be used to closely reproduce the more complex gene expression patterns [28]. When this study was started, cDNA microarrays were not used yet as a standard of classification for clinical practice and design of therapeutic strategy. We utilized the immunohistochemical classification scheme, adopting the terminology of triple negative to characterize patients with ER−, PGR−, HER2−, at high risk for recurrence [29]. Here, we present the 81 months follow-up of a study, of patients with TNBC, treated homogeneously by a multi-modality therapeutic protocol that included induction therapy with taxanes and anthracyclines, surgery, simultaneous CMF and XRT, and two courses of high-dose CBDCA, VP-16, ifosfamide, supported by hematological growth factors. Several reports focusing on the treatment of TNBC have confirmed the worse prognosis of this cancer type, compared with others. Hence, despite the initial sensitivity and high response rate to chemotherapy, patients with TNBCs show a paradoxical feature with poor long-term outcome [9]. The largest described series from MD Anderson Hospital [30] included a cohort of 1118 patients who had received neoadjuvant chemotherapy. Of these patients, 255 (23%) had TNBC. Patients with TNBC compared with non-TNBC had significantly higher pCR rates but lower 3-year PFS and OS rates.

Other strategies have been adopted to improve the clinical outcome of patients with TNBC. In recent years several studies have emphasized the role of vascular endothelial growth factor (VEGF) as a key mediator of angiogenesis. Bevacizumab (Avastin), the best-known anti-angiogenic agent, is a humanized monoclonal antibody that binds to VEGF and prevents it from interacting with vascular endothelial cells [31, 32]. Bevacizumab has been shown to improve response rate and PFS when combined with chemotherapy in patients with TNBC. However, a 2011 meta-analysis highlighted the dangers of the drug: Compared with chemotherapy alone, the addition of bevacizumab was associated with an increased risk of fatal adverse events, the most common being hemorrhage (23.5%), neutropenia (12.2%), and gastrointestinal tract perforation (7.1%), especially when bevacizumab was associated with taxanes or platinum drugs [33]. TNBC have intrinsic defects in mechanisms of DNA repair, making this cancer a rational target for therapy based on PARP inhibition. In a phase II study, a total of 123 patients were randomly assigned to receive gemcitabine and carboplatin with or without the PARP inhibitor, Iniparib. The addition of Iniparib to chemotherapy improved the clinical benefit and survival of patients with metastatic TNBC without significantly increased toxicity [8].

We and others have previously shown that two courses of CBDCA, CTX, and VP16 as consolidation therapy provide significant survival benefits to patients with basal-like TNBC [22, 34]. Gluz et al. [35], in a randomized trial, evaluated a cohort of patients with high-risk breast cancer, with >9 involved lymph nodes, who received different chemotherapy dose-intensification strategies. The most pronounced benefit of dose-intensified chemotherapy was observed in triple-negative tumors: In this subgroup, median PFS was not reached in the high-dose arm, whereas it was only 32.3 months in the dose-dense arm. This translated into an estimated 5-year PFS of 71% in the triple-negative cohort treated by high-dose compared with only 26% in the dose-dense arm.

Collectively, it is common opinion that mastectomy renders radiation therapy unnecessary unless the tumor is 5 cm or larger, its margins are irregular with signs of invasion through basal membranes of adjacent normal mammary tissue, or if there is nodal metastasis. Tseng et al., however, suggested that adjuvant radiation in all patients with metaplastic breast cancer may lead to improved OS [36]. Due to the high locoregional recurrence rate that TNBC shows, we selected to administer concurrent radiation therapy with CMF-based chemotherapy, a strategy whose rationale is supported by recent studies demonstrating that the magnitude of benefit of CMF chemotherapy is largest in patients with triple-negative, node-negative breast cancer [37].

In conclusion, in our cohort of patients with TNBC our multipronged approach involving induction chemotherapy, followed by chemoradiation therapy and by HDCT provides a prolonged disease-free period and a significant increase in OS, with an acceptable toxicity profile. We anticipate that our results will prompt the application of our treatment strategy in the clinical management of TNBC.
